# The 2016 Ming K Jeang Award for Excellence in *Cell & Bioscience*

**DOI:** 10.1186/s13578-017-0169-z

**Published:** 2017-08-10

**Authors:** Yun-Bo Shi

**Affiliations:** The National Institutes of Health, Bethesda, MD USA

## Abstract

Two research groups led by Dr. Jim Hu of University of Toronto, Canada and Dr. Renping Zhou of Rutgers University, USA, respectively, won the 2016 Ming K Jeang Award for Excellence in *Cell & Bioscience.*

## Editorial

We are very pleased to announce that two research groups, who each published an outstanding research article in *Cell & Bioscience* in 2016, have been selected to receive the Ming K Jeang Award for Excellence in *Cell & Bioscience*. The Ming K Jeang Award for Excellence in *Cell & Bioscience* was established in 2011 with a generous donation from the Ming K. Jeang Foundation to honor outstanding research articles published in *Cell & Bioscience*, the official journal of the Society of Chinese Bioscientists in America (SCBA; http://www.scbasociety.org). A committee of *Cell & Bioscience* Editors, chaired by Dr. Dong-Yan Jin, considered all research articles published in the journal in 2016 to select the following two articles to receive the award [1, 2]:

## Epithelium-specific Ets transcription factor-1 acts as a negative regulator of cyclooxygenase-2 in human rheumatoid arthritis synovial fibroblasts

Chan-Mi Lee, Sahil Gupta, Jiafeng Wang, Elizabeth M. Johnson, Leslie J. Crofford, John C. Marshall, Mohit Kapoor and Jim Hu.

Cell & Bioscience 2016 6:43.

## EphA5 and EphA6: regulation of neuronal and spine morphology

Gitanjali Das, Qili Yu, Ryan Hui, Kenneth Reuhl, Nicholas W. Gale and Renping Zhou.

Cell & Bioscience 2016 6:48.

Congratulations to these two groups of investigators for jobs well done!

We are looking forward to receiving contributions of outstanding research articles from the scientific community in 2017 and beyond.
